# Patient With a 42-Year History of Coccidioidal Meningitis

**DOI:** 10.1177/2324709618820047

**Published:** 2018-12-20

**Authors:** Carlos D’Assumpcao, Arash Heidari, Royce H. Johnson

**Affiliations:** 1 Kern Medical—UCLA, Bakersfield, CA, USA; 2 Ross University, Miramar, FL, USA; 3 Valley Fever Institute, Bakersfield, CA, USA

**Keywords:** extrapulmonary coccidioidomycosis, disseminated coccidioidomycosis, coccidioidal meningitis

## Abstract

This is a case of a 56-year-old man diagnosed with coccidioidal meningitis 42 years ago at the age of 14. He was treated with intrathecal amphotericin B deoxycholate by cisternal puncture for 15 years before switching to fluconazole once it became available in 1991. Over 42 years of treatment, he developed hearing loss due to auditory nerve neurotoxicity, hydrocephalus requiring ventriculoperitoneal shunting with associated malfunctions, lumbar arachnoiditis, and hypokalemic paralysis. Regular cerebrospinal fluid studies to this day do not show disease clearance. Many of the lessons from his clinical history are enshrined in the current iteration of the Infectious Diseases Society of America Coccidioidomycosis Treatment Guidelines. To our knowledge, he is the longest surviving coccidioidal meningitis patient.

## Introduction

Meningitis is the most feared form of extrapulmonary coccidioidomycosis, which is caused by fungal species *Coccidioides immitis* or *Coccidioides posadasii*.^1^ In the 2016 Infectious Diseases Society of America Clinical Practice Guideline for the Treatment of Coccidioidomycosis, the treatment of choice is 400 to 1200 mg oral fluconazole. If this clinically fails, options are to change to another azole or to initiate intrathecal amphotericin B therapy. For the most common complication, hydrocephalus, a shunt for decompression is nearly always required.^2^ Reoccurrences after presumed “cure” at 3 years have been reported^3,4^; thus, duration of therapy is now considered to be lifelong.

## Case

A 54-year-old Caucasian man was initially diagnosed with central nervous system coccidioidomycosis at age 14 in 1976. He received intrathecal amphotericin B deoxycholate on a declining schedule from age 14 to 29 via cisternal puncture. He suffered auditory, but not vestibular, nerve damage related to intrathecal amphotericin B deoxycholate neurotoxicity.^5^

After fluconazole was approved by the US Food and Drug Administration in early 1991,^6^ he was started on fluconazole 400 mg. Mild hydrocephalus was initially detected at this point. The hydrocephalus continued to worsen, and by 1995, at age 31, a ventriculoperitoneal shunt was placed, which required 2 subsequent revisions.

He had persistent cerebrospinal fluid (CSF) cultures of *Coccidioides*, and therefore, fluconazole was gradually increased up to his current dose of 1200 mg by 2001.^7^

In 2008, at age 45, he developed severe lumbar pain and was found to have lumbar arachnoiditis. Soon thereafter, he developed a neurogenic bladder and suffered from erectile dysfunction. As a result, CSF monitoring was returned to cisternal puncture due to the lumbar pain. He had a hypokalemic paralysis that was suspected to be related to fluconazole therapy in 2011, at age 48. Most recently, in 2017, he had an episode of headache and ataxia secondary to transient ventriculoperitoneal shunt malfunction.

After an episode of unstable angina in 2010, at age 47, he underwent a heart catheterization that demonstrated coronary artery disease leading to a quadruple coronary artery bypass. In January 2016, at age 53, he suffered a non-ST elevation myocardial infarction for which he received multiple drug-eluting stents, and he was started on dual antiplatelet therapy. His current medications are lisinopril, metoprolol, aspirin, clopidogrel, ezetimibe, anti-PCSK9 monoclonal antibody bimonthly injections (evolocumab), and fluconazole 1200 mg daily.

**Figure 1.**
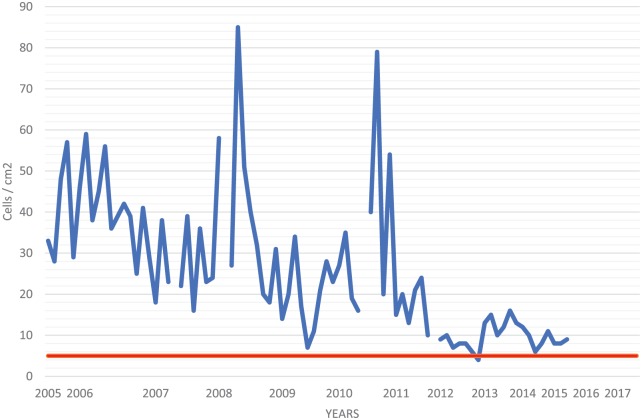
Cisternal cerebrospinal fluid (CSF) white blood cell count. CSF analyses were conducted every 1 to 3 months. Normal limit: <5 cells/cm^2^ (red line).

**Figure 2.**
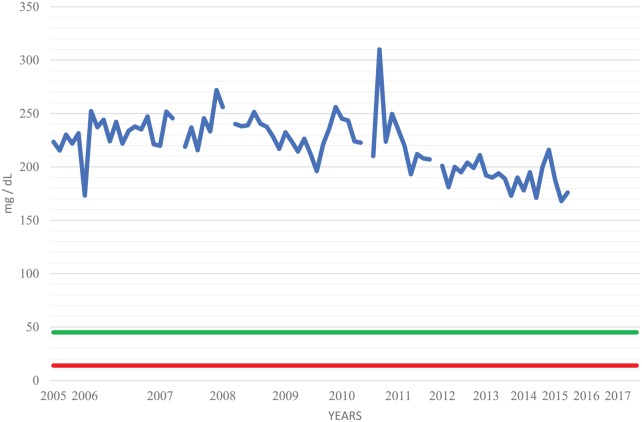
Cisternal cerebrospinal fluid (CSF) protein. CSF analyses were conducted every 1 to 3 months. Normal values: 15 (red) to 45 (green) mg/dL.

**Figure 3.**
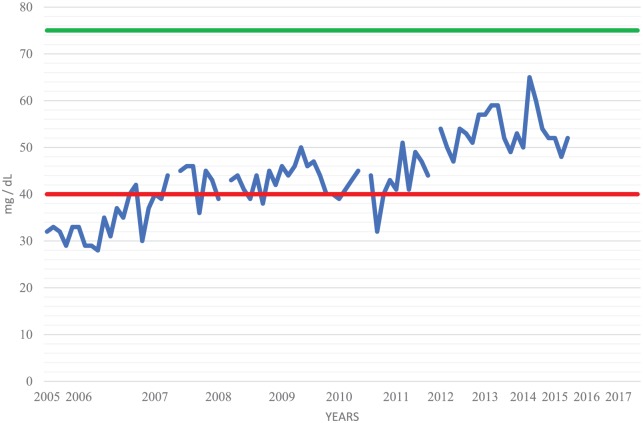
Cisternal cerebrospinal fluid (CSF) glucose. CSF analyses were conducted every 1 to 3 months. Normal values: 40 (red) to 75 (green) mg/dL.

**Figure 4.**
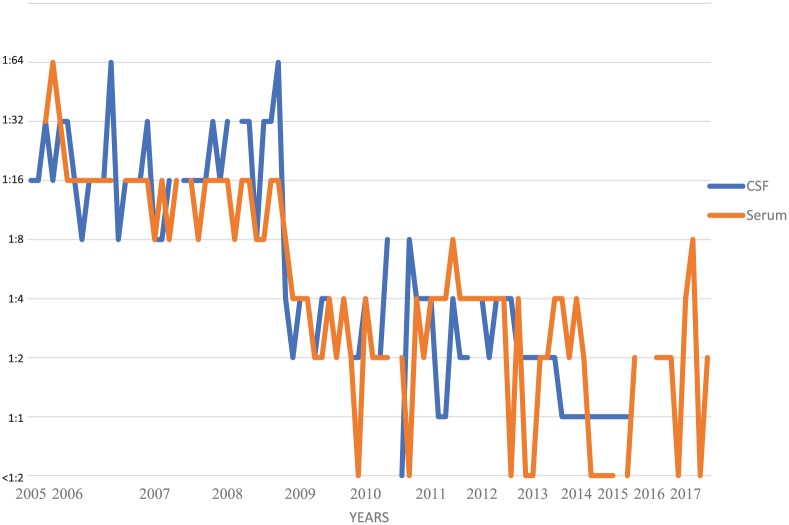
Serological complement fixation titers. Cerebrospinal fluid analyses were conducted every 1 to 3 months.

**Figure 5.**
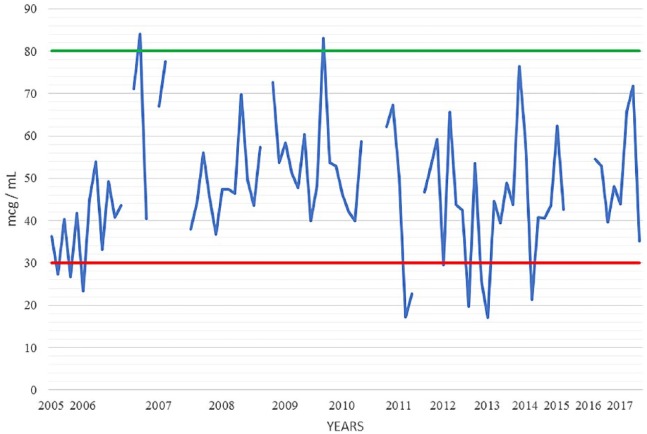
Serum fluconazole level. Cerebrospinal fluid analyses were conducted every 1 to 3 months. Currently, there is no universally accepted therapeutic serum level. At Kern Medical, the Division of Infectious Diseases accepts serum fluconazole levels of 30 (red) to 80 (green) µg/mL.^11^

Despite 42 years of therapy, longitudinal CSF studies reveal persistent lymphocytic pleocytosis (Figure 1), elevated protein (Figure 2), normalizing glucose (Figure 3), and persistent complement fixation titers (Figure 4). The clopidogrel represents a contraindication to cisternal puncture; therefore, there are no recent CSF studies. Last available CSF studies in late 2015 showed white blood cell count of 13 cells/cm^2^ (89% lymphocytes, 11% monocytes), protein 170.5 mg/dL, glucose 45 mg/dL, and immunoglobulin G (IgG) immunodiffusion reactivity with complement fixation titer of 1:1. The most recent serum IgG immunodiffusion was reactive with complement fixation titer as high as 1:8. Serum fluconazole levels have been routinely collected (Figure 5). The patient refused to try a newer azole.

## Discussion

Patients with untreated central nervous system coccidioidomycosis have 100% mortality 2 years after initial diagnosis.^8^ Even with aggressive treatment, 30% of patients may still die early in the disease course.^1,9,10^ Seventy percent of patients have treatment response and improve to varying degrees but are on lifelong therapy.^11^ This patient is living symbiotically with *Coccidioides*.

This patient has been clinically stable since his last CSF analysis in 2015. He has been adherent to high-dose fluconazole therapy. Based on available data since 2005, despite serum fluconazole drug levels that were largely within accepted limits (Figure 5) as per current practice of his infectious disease physicians,^11^ he still has persistent lymphocytic pleocytosis, elevated protein, reactive IgG immunodiffusion, and positive complement fixation titer. To our knowledge, he is the longest surviving patient with CNS coccidioidomycosis. The need for indefinite lifelong high-dose azole suppressive therapy is demonstrated here.
